# Tolerability and Outcomes for Treatment of Older Myxoid Liposarcoma Population

**DOI:** 10.3390/cancers16183233

**Published:** 2024-09-23

**Authors:** Reilly A. Coombs, Judith Jebastin Thangaiah, Brittany L. Siontis, Steven I. Robinson, Scott H. Okuno, Matthew T. Houdek, Meng Xu-Welliver, Thanh P. Ho

**Affiliations:** 1 School of Medicine, Medical College of Wisconsin-Central Wisconsin, Wausau, WI 54401, USA; 2 Department of Research and Innovation, Mayo Clinic Health System, Rochester, MN 55905, USA; 3 Department of Anatomic Pathology, Mayo Clinic, Rochester, MN 55905, USA; 4 Department of Oncology, Division of Medical Oncology, Mayo Clinic, Rochester, MN 55905, USA; 5 Department of Orthopedic Surgery, Mayo Clinic, Rochester, MN 55905, USA; 6 Department of Radiation Oncology, Mayo Clinic, Rochester, MN 55905, USA

**Keywords:** myxoid liposarcoma, soft tissue sarcoma, older patients

## Abstract

**Simple Summary:**

Myxoid liposarcoma (MLPS) is a rare soft tissue sarcoma, often affecting the proximal extremity. There is currently limited literature describing MLPS in older individuals. This retrospective study describes treatment outcomes and tolerability in older patients treated for non-metastatic MLPS. The study shows that older patients with MLPS tolerated systemic therapy, radiation therapy, and surgery with few toxicities.

**Abstract:**

Background: Myxoid liposarcoma predominantly affects young and middle-aged individuals, and little is known regarding treatment tolerability and outcomes in older patients. This study aims to better understand this older patient population. Methods: This single institution retrospective study included patients aged 70 years and older with localized (non-metastatic) myxoid liposarcoma. Results: Sixteen patients were included. The median age was 75 years, and 9 (56%) were female. Fourteen (88%) were extremity tumors and two (12%) were trunk. The median tumor size was 10.4 cm (range, 3.6 to 28 cm). Five (31%) tumors had a round cell component. All patients had surgery. Fourteen (88%) had perioperative radiation, and three (19%) had perioperative chemotherapy. One patient had postoperative infection, and one patient had neutropenic fever from preoperative chemotherapy. The median follow up from surgery was 6.3 years. Eight (50%) patients died from MLPS. The median relapse-free survival and overall survival were 34 months and 75 months, respectively. Conclusions: Most older patients with localized MLPS received perioperative radiation therapy with surgery, and few serious toxicities were reported. Even with treatment, half of the patients relapsed.

## 1. Introduction

Myxoid liposarcoma (MLPS) is a rare type of cancer, accounting for approximately 5% of all soft tissue sarcoma in adults [1]. It typically affects the deep proximal extremities, such as the thighs or upper arms. Unlike other liposarcomas, the retroperitoneum is a rare primary site for MLPS. The molecular hallmark of MLPS is the presence of specific translocations which can help confirm diagnosis: most commonly, FUS-DDIT3 fusion, and less frequently, EWSR1-DDIT3 fusion [1,2]. Unlike other translocation associated sarcomas, these MLPS fusion transcripts are not associated with prognosis [1,3]. 

Local relapse is not common in MLPS, but patients can develop distant metastasis months or even years after treatment. Studies have reported that between 30% to 60% of patients with MLPS develop metastases [1]. In addition to lung metastases, MLPS can develop metastasis to unusual locations such as bone (particularly spine) and soft tissue [4]. A key prognostic factor is the round cell component of MLPS. Tumors with a greater than 5% round cell component are considered high grade myxoid liposarcoma, and they are associated with a significantly higher risk of metastasis and death [5,6]. MLPS survival is superior to other sarcoma subtypes, with a 5-year overall survival of 78% to 91% reported, although lower rates of survival have been reported for patients with metastatic disease at diagnosis and tumors with round cell or high grade features [3,6,7].

In comparison to other soft tissue sarcomas, MLPS is considered sensitive to both radiation therapy and chemotherapy. Radiation therapy can improve local control of tumors that are resected [8,9]. Response rates to doxorubicin-based chemotherapy regimens are approximately 40%, and trabectedin shows a response rate of approximately 20% to 50% [10,11,12,13]. Given the response rate in MLPS, doxorubicin and trabectedin have been evaluated in prospective trials for treatment of localized MLPS, including a randomized trial showing non-inferiority of trabectedin compared to an anthracycline/ifosfamide combination in a neoadjuvant setting [14,15].

For many subtypes of soft tissue sarcoma, older age can be associated with lower survival and higher risk of recurrence [16]. MLPS is less frequently diagnosed in older patients, but age appears to be an important prognostic factor [17,18]. In older patients, age can also be a factor in deciding whether to proceed with any treatment [19]. This study aims to evaluate the tolerability of treatment in older patients with localized MLPS and to assess their outcomes.

## 2. Materials and Methods

This retrospective multi-site study was approved by the Mayo Clinic Institutional Review Board (Rochester, MN, USA) and complies with the Health Insurance Portability and Accountability Act (HIPAA). Thirty-three patients were initially identified from the Mayo Clinic Electronic Medical Database who were diagnosed with non-metastatic myxoid liposarcoma between 1992 to 2021. There is no chronologic age that defines an older adult with cancer [20]. For this study, the age of 70 years and older was chosen as the cutoff to identify patients who are potentially vulnerable to treatment toxicities since many geriatric assessments focus on adults in this age range [20,21]. Their charts were reviewed to collect patient demographics, treatment course, and follow up. Patients with missing clinical notes or notes that lack treatment course, patients who did not undergo treatment, or patients with less than two years of follow up were excluded from analysis. High grade disease on pathology was identified as having a round cell component and/or grade 3 and higher. Seventeen patients were excluded from analysis. Specifically, among the excluded patients were nine patients who did not undergo treatment for their MLPS, seven patients who had missing clinical notes, and one patient who in the chart review actually had metastasis at the time of MLPS diagnosis. Ultimately, sixteen patients aged 70 years and older who were treated for localized MLPS were included in the study. Descriptive statistics, relapse-free survival, and overall survival were calculated using Microsoft Excel Version 2403 (Redmond, WA, USA). A Cox proportional hazards regression model was calculated using IBM SPSS Statistics version 29.0.2.0 (Armonk, NY, USA).

## 3. Results

A total of sixteen patients with myxoid liposarcoma were included (See Table 1). The median age was 75 years (range, 70–85 years) at the time of diagnosis and the majority of patients were female (9 patients, 56%). All patients identified as non-Hispanic White. The median body mass index (BMI) of patients prior to surgery was 32 (range, 22.9–45.2). Seven (44%) patients in this cohort were either current or former smokers. Many patients had medical comorbidities: cardiac (hypertension (10 patients), hyperlipidemia (4 patients), coronary artery disease (4 patients), dilated cardiomyopathy (1 patient), systolic heart failure (1 patient)); endocrine (diabetes/impaired glucose intolerance (4 patients), hypothyroidism (1 patient), osteoporosis (3 patients)); gastrointestinal (gastroesophageal reflux disease (3 patients)); renal (chronic kidney disease (1 patient), nephrolithiasis (1 patient)); neurologic (dementia (2 patients), Parkinson disease (1 patient)); and infectious (tuberculosis (1 patient)). 

Fourteen (88%) patients had extremity tumors (3 upper extremity, 11 lower extremity) and two (12%) patients had trunk tumors. Five tumors (31%) had a round cell component (percentage was not reported). All patients had had surgery for the primary tumor. The median size of the resected tumor was 10.4 cm (range, 3.6 to 28 cm). Fifteen (94%) had R0 resection, and one (6%) had R1 resection (unplanned). Most patients recovered well from surgery. One (6%) patient developed a postoperative infection that was treated with irrigation/debridement and antibiotics with resolution of the infection. No surgical complications were reported for the other patients. The median Eastern Cooperative Oncology Group (ECOG) performance status of this cohort was 1 (range, 0–4) postoperatively. 

Fourteen (88%) patients received perioperative radiation therapy. Five (36%) of these patients had preoperative radiation therapy and nine (64%) of these patients had postoperative radiation therapy. Two (14%) of these patients had preoperative radiation therapy in addition to intraoperative radiation therapy. The radiation therapy dose ranged from 5000 to 6480 centigray (cGy). Patients tolerated radiation therapy well and reported minimal side effects from radiation therapy. The most commonly reported adverse events included: fatigue, decreased appetite, gastrointestinal side effects, and erythema at the radiation site. No long-term toxicities were reported related to radiation therapy.

Three (19%) patients with high grade disease received perioperative chemotherapy. In Case 1, the patient received two cycles of ifosfamide/etoposide preoperatively; this patient was hospitalized for neutropenic fever and chemotherapy was discontinued. The patient in Case 11 completed 3 cycles of doxorubicin/ifosfamide postoperatively, and the patient in Case 13 had preoperative cisplatin with concurrent preoperative radiation. Both patients in Case 11 and 13 completed perioperative chemotherapy without serious adverse events. Additional patient details are noted in Table 2.

In this cohort, half (8, 50%) of the patients had MLPS relapse. One patient (6%) had both local and distant metastasis, and the remainder of the patients had distant metastasis. The location of metastasis included bone (7), lung (4), and liver (1). Of the eight patients with recurrence, three (38%) had primary tumors with round cell, and seven (88%) had primary extremity tumors. The patient with local recurrence had additional surgery with negative margins; otherwise, no patients had surgery for metastatic sites. Five patients (63%) had radiation therapy, and five patients (63%) had systemic therapy for relapse. Palliative systemic therapies included: Case 1 (doxorubicin/olaratumab, trabectedin, gemcitabine), Case 3 (mitomycin C/doxorubicin/cisplatin), Case 8 (nivolumab), Case 10 (doxorubicin), and Case 11 (doxorubicin/ifosfamide). The patient in Case 1 developed multiple side effects from trabectedin including diarrhea, nausea, vomiting, dehydration, and subsequent acute kidney injury. Due to these toxicities, the patient switched to gemcitabine, which was discontinued due to significant anemia and acute kidney injury. The anemia improved and the acute kidney injury resolved after chemotherapy discontinuation. Otherwise, patients receiving palliative systemic therapy reported minimal side effects including fatigue, anorexia, and emesis most commonly. 

Patients had a median follow up of 6.3 years (range: 5 months to 14.7 years). Eight patients (50%) died from MLPS. The median relapse-free survival and overall survival was 34 months (range, 5 to 76 months) and 75 months (5 to 176 months), respectively. The five-year local recurrence-free and metastasis-free survival was 94% and 69%, respectively. Three of the five patients who had tumors with a round cell component died from MLPS. 

A Cox proportional hazard regression analysis showed that primary tumors larger than 10 cm were significantly associated with an increased hazard of recurrence compared to smaller tumors (hazard ratio = 0.1755, *p*-value = 0.003). In contrast, high grade and tumor location (extremity versus trunk) did not show significant association with recurrence risk. Patient factors such as older age (above 80 years), more comorbidities (2 or more), and BMI (above 25) did not show significant association with recurrence. 

## 4. Discussion

Similar to the clinical presentation observed in younger individuals, our study demonstrates that myxoid liposarcoma can present as a large tumor in the proximal extremities of older adults. In our study, nearly 90% of older patients diagnosed with MLPS underwent surgical resection with perioperative radiation therapy. Over 90% of patients had complete resection of the primary tumor with negative margins. This approach of radiation therapy and resection has been shown to be of benefit for local control of MLPS and is supported by European and American guidelines for treatment of localized soft tissue sarcoma [8,22,23]. The DOREMY trial demonstrated the safety and efficacy of a shorter course of preoperative radiation therapy (36 Gy instead of 50 Gy) in localized MLPS, which has become an appealing option for many patients [24]. Importantly, adverse events related to surgery or radiation therapy were relatively uncommon among older patients in this study, suggesting they are generally able to tolerate radiation therapy and surgery with few significant toxicities. A similar finding was observed in a Japanese study of patients aged 80 years and above who had few complications with curative intent surgery for soft tissue sarcoma [25]. The Japanese study concluded that surgery should be considered in older patients. In contrast to the common use of perioperative radiation therapy in our study of older patients, a large population-based study of Dutch patients with a median age of about 50 years showed that only about half of the patients with localized MLPS received perioperative radiation therapy [17]. The Dutch study also showed that most patients received postoperative radiation therapy compared to preoperative radiation, which is similar to our study’s finding. These differences may be reflective of historical treatment preferences, evolving guidelines over time, and possible differences in clinical decision-making based on individual patient factors. Notably, fewer than 20% of patients in our study received perioperative chemotherapy, and the ones who received it had high grade disease. Only one patient received doxorubicin/ifosfamide, even though anthracycline/ifosfamide is considered the standard of care perioperative regimen for high-risk, localized soft tissue sarcoma based on a randomized phase III trial of neoadjuvant chemotherapy [26]. This may reflect guidelines that perioperative chemotherapy should be considered on a case-by-case basis and not routinely recommended as a standard of care treatment for non-metastatic soft tissue sarcoma [22,23]. Specifically, perioperative chemotherapy can be associated with significant toxicities but may have limited benefit in improving local control or overall survival.

In this cohort of adults aged 70 and above, the incidence of local relapse was low, with only 6% of patients experiencing a recurrence of MLPS at the original site of the disease. This rate is notably lower than local relapse reported in other published studies, in which local recurrence ranged from 9% to 25% [1,27]. By comparison, half of the patients in our study developed distant metastatic disease within 6 years of treatment. Approximately 60% of patients with relapsed disease were treated with palliative systemic therapy or radiation therapy, even though MLPS is considered radiosensitive and chemosensitive (especially to doxorubicin and trabectedin). More specifically, although four patients (25%) received doxorubicin-based chemotherapy, only one patient (6%) received trabectedin in our study. This may reflect a combination of factors, including concerns from providers and patients about the potential toxicities of therapies, the presence of other comorbidities that could affect treatment tolerability, and the impact on the patient’s quality of life. Of the patients who received systemic therapy for relapsed disease, only one patient had more than one line of palliative systemic therapy. This was a similar finding to a Greek retrospective study of older patients with advanced soft tissue sarcoma. The Greek study showed that patients age 75 years and above were less likely to receive treatment compared to younger patients; in addition, for older patients who did receive therapy, they were more likely to receive single agent therapy and a lower dose intensity [28]. Some of the systemic therapies administered to the patients in our study are not commonly used in current clinical practice to treat soft tissue sarcoma, highlighting the advancement of treatment over time. For instance, doxorubicin/olaratumab is no longer used for soft tissue sarcoma, after a randomized phase III trial showed no survival benefit with addition of olaratumab [29]. Ongoing studies will hopefully provide more therapeutic options for patients with advanced disease, notably by investigating novel combination therapies, such as immunotherapy and vaccines [30]. Some of these therapies, however, can be associated with unique toxicities. For instance, afamitresgene autoleucel is a cell therapy which was demonstrated to be of potential benefit for select individuals with myxoid liposarcoma but was associated with significant risk of cytokine release syndrome in a phase II trial [31]. Our study shows that serious adverse events were few, suggesting favorable tolerability in this older patient cohort. This has also been observed in another study showing few adverse events in older patients who received chemotherapy, although the survival benefit with treatment was limited [28]. This highlights that metastatic disease in MLPS, like other soft tissue sarcomas, can be difficult to treat and ultimately lead to death. 

In our study, older patients who initially presented with localized disease had a 5-year overall survival rate of 63%. Tumors that were greater than 10 cm were associated with worse survival in our study, which is consistent with findings from other studies [8,17]. The survival rate is lower than those reported in other studies, in which 5-year overall survival rates of 80% or greater have been documented [17,18,27]. These findings underscore the complexity of treating MLPS in older adults, whereby balancing therapy options with the patient’s overall well-being can be a challenge. An important consideration for shared decision-making in this patient population is the utilization of geriatric screening tools and comprehensive assessments, which are part of European and American guidelines for care of older patients with cancer [20,32,33]. These tools and assessments can help identify patients who may be at risk for complications from treatment and who may benefit from additional supportive measures. Geriatric assessment can significantly reduce high grade adverse events related to chemotherapy and can be a cost-effective strategy in treating older patients with curative intent therapy [34,35]. By tailoring treatment approaches to the specific needs and health status of older patients, clinicians may enhance patient quality of life although the potential benefit to survival is unknown. Our study underscores the importance of considering age as one of many factors in the treatment of MLPS and the need for ongoing research to optimize therapeutic strategies for older adults.

This study has limitations relating to the historical nature of the retrospective study design. Minor adverse events may not have been documented in clinical notes, although serious adverse events such as hospitalizations were reported. Changes to performance status was not routinely documented in clinical notes, and this may have impacted treatment decisions and patient outcomes. Poor performance status, for instance, has been associated with the worst overall survival in older patients with soft tissue sarcoma and has been associated with mortality risk in geriatric screening tools [36,37]. Most patients received perioperative radiation therapy and few patients received perioperative chemotherapy for localized MLPS, precluding meaningful analysis of the treatment effect and survival. Although some treatment approaches have changed in the study’s 30-year interval, perioperative radiation therapy with resection remains the consistent standard of care. In addition, this extended interval was needed to capture the long-term follow up for a rare diagnosis which is less commonly diagnosed in the older patient population.

## 5. Conclusions

In this cohort of patients aged 70 years and above with localized myxoid liposarcoma, the majority received perioperative radiation therapy with surgery. For patients who developed relapse, radiation therapy and systemic therapy were more commonly used compared to surgery. Few patients experienced serious adverse events from local or systemic therapies, although survival was lower in this older cohort, highlighting the challenges that age related factors might contribute to the treatment of MLPS. Utilization of geriatric screening may help clinicians identify older patients who may be at risk for complications from MLPS treatment and might benefit from additional support. 

## Figures and Tables

**Table 1:** Summary of older patients with myxoid liposarcoma.

	Extremity (n = 14)	Trunk (n = 2)	Total (n = 16)
Female, n (%)	8 (57%)	1 (50%)	9 (56%)
Median age, years (range)	76 (70–85)	70.5 (70–71)	75 (70–85)
Median tumor size, cm	10.4 (3.6–28.0)	10.8 (10.0–11.5)	10.4 (3.6–28.0)
Surgical margin	R0: 13 (93%) R1: 1 (7%)	R0: 2 (100%)	R0: 15 (94%) R1: 1 (6%)
Recurrence, n (%)	7 (50%)	1 (50%)	8 (50%)
Median time from surgery to recurrence, months (range)	47 (5–76)	15	33.5 (5–76)
Surgery for recurrence, n (%)	0 (0%)	1 (100%)	1 (13%)
Radiation therapy for recurrence, n (%)	5 (71%)	0 (0%)	5 (63%)
Systemic therapy for recurrence, n (%)	4 (57%)	1 (100%)	5 (63%)

**Table 2:** Outcomes of older patients with myxoid liposarcoma.

Case	Age	Sex	Site	Size (cm)	Grade	Resection	Radiation Therapy (Dose, If Available)	Perioperative Chemotherapy	Metastasis	Outcome
1	74	F	E	8.5	G3, RC	R0	Postop (64 Gy)	Ifosfamide/etoposide (2 cycles, preop)	Bone, 47 mos	DOD, 68 mos
2	75	M	E	7.3	G4, RC	R0	Postop (50 Gy)	None	Liver and bone, 5 mos	DOD, 5 mos
3	76	M	E	8.0	G4, RC	R0	Postop (64 Gy)	None	Lung and bone, 14 mos	DOD, 42 mos
4	73	M	E	5.5	G2	R1	Preop	None		DUC, 109 mos
5	85	F	E	4.4	G1	R0	None	None		DUC, 176 mos
6	79	F	E	10.2	NA	R0	Preop (62.5 Gy), IORT (10 Gy)	None	Spine and occipital bone, 20 mos	DOD, 28 mos
7	76	M	E	11.5	G3, RC	R0	Postop (63 Gy)	None		NED, 55 mos
8	70	F	E	20.8	G2	R0	Preop (50 Gy)	None	Lung and spine, 76 mos	DOD, 108 mos
9	74	F	E	14.0	G1	R0	None	None		NED, 123 mos
10	78	F	E	12.5	G2	R0	Postop	None	Thigh, pelvis, and retroperitoneum, 76 mos	DOD, 79 mos
11	70	M	T	10.0	G3	R0	Postop (64.8 Gy)	Doxorubicin/ifosfamide (3 cycles postop)	Local relapse, 15 mos. Lung and pleura, 22 mos	DOD, 30 mos
12	80	M	E	10.5	G1	R0 (postoperative infection)	Preop (50 Gy)	None		NED, 12 mos
13	71	F	T	11.5	G4	R0	Preop (50 Gy), IORT	Cisplatin (with concurrent preop radiation therapy)		U, 129 mos
14	77	F	E	28.0	G1	R0	Postop	None		NED, 79 mos
15	70	F	E	17.8	G3, RC	R0	Postop	None		U, 144 mos
16	84	M	E	3.6	G2	R0	Postop (50 Gy)	None	NA, 68 mos	DOD, 71 mos

Abbreviations: DOD—died of disease, DUC—died of unrelated cause, E—extremity, F—female, M—male, mos—months, NA—not available, preop—preoperative, postop—postoperative, NED—no evidence of disease (last day of follow up), RC—round cell, T—trunk, U—unknown cause of death.

## Data Availability

Due to privacy and ethical considerations, the data is not publicly available. Requests for data access should be directed to the corresponding author.
